# Natural Products Targeting Immune Mechanisms in Ocular Inflammation: Uveitis and Dry Eye

**DOI:** 10.3390/cimb48040367

**Published:** 2026-04-01

**Authors:** Wenjia Lu, Mingming Yang, Yaru Zou, Jing Zhang, Kyoko Ohno-Matsui, Koju Kamoi

**Affiliations:** Department of Ophthalmology & Visual Science, Graduate School of Medical and Dental Sciences, Institute of Science Tokyo, Tokyo 113-8510, Japan; lucern1wj@outlook.com (W.L.); yangmm-12@outlook.com (M.Y.); alicezouyaru519@gmail.com (Y.Z.); zhangj.c@foxmail.com (J.Z.); k.ohno.oph@tmd.ac.jp (K.O.-M.)

**Keywords:** uveitis, dry eye disease, natural products, inflammatory cytokines, immune mechanisms, cytomegalovirus

## Abstract

At present, the clinical management of ocular inflammatory diseases predominantly relies on chemically synthesized therapeutic agents. Although these therapies demonstrate established efficacy, their long-term use is associated with substantial economic burden. In addition, they may cause ocular side effects and systemic adverse reactions involving the cardiovascular, hepatic, and renal systems. In contrast, natural products have attracted increasing attention in recent years because many are accessible, relatively cost-effective, and potentially well tolerated. Studies indicate that various natural products exert anti-inflammatory and immunomodulatory effects by inhibiting inflammatory signaling pathways such as NF-κB and MAPK, regulating immune cell function and alleviating oxidative stress responses. These multifunctional properties support their potential therapeutic value in various inflammatory diseases. Notably, several natural products have shown potential benefits in clinical trials; however, their investigation and application in ocular diseases remain relatively limited. In this review, we focus on uveitis and dry eye disease (DED) as representative ocular disease models and systematically summarize the current research progress on four natural products—*Paeonia lactiflora* extracts, resveratrol and its derivatives, curcumin, and boswellic acids in experimental studies of ocular diseases. We particularly focus on their effects in alleviating ocular surface inflammation and intraocular inflammatory responses through their immunomodulatory mechanisms. This review aims to provide a mechanistic framework for understanding the potential role of natural products as complementary or alternative strategies to current therapeutic approaches, while informing the development of novel therapeutics and future research directions in ocular diseases.

## 1. Introduction

Ocular inflammatory diseases constitute a significant cause of visual dysfunction, including a broad spectrum of disorders affecting both ocular surface and intraocular tissues. Among these, uveitis and DED are two representative ocular inflammatory diseases [1,2]. Uveitis is characterized as a complex immune-mediated disease within intraocular tissues [3,4], whereas DED is a chronic inflammatory condition of the ocular surface associated with tear film instability [1,5]. Accumulating evidence indicates that although these two inflammation-related ocular diseases have distinct etiological factors, they share similar immunopathological mechanisms, involving multiple inflammatory signaling pathways, aberrant regulation of macrophage and effector T cell subsets alongside cytokines [6,7,8]. Among these, the excessive expansion of Th1 and Th17 cells has been recognized as a critical immunological basis for persisting chronic inflammation [1,8,9,10,11,12].

Current therapeutic strategies for ocular inflammatory diseases include anti-infective therapy for infectious etiologies and anti-inflammatory/immunomodulatory therapy for immune-mediated inflammation. However, long-term disease control and safety remain challenging; the prolonged use of conventional therapies is often associated with ocular or systemic side effects, reduced patient compliance, and high treatment costs, all of which limit their long-term clinical utility [2,13,14,15]. Taken together, these findings underscore the necessity for developing safer alternative therapeutic strategies that specifically target immunopathological pathways.

Natural products have emerged as promising candidates for immune-mediated diseases because of their multi-target, anti-inflammatory and immunomodulatory properties [16,17]. However, their immunomodulatory roles in ocular inflammatory diseases have not been fully illustrated. In particular, systematic analyses linking immune mechanisms to therapeutic outcomes in uveitis and DED are still lacking.

In this review, we summarize recent experimental evidence on *Paeonia lactiflora* extracts, resveratrol and its derivatives, curcumin, and boswellic acids, with a particular focus on their immune-related mechanisms in uveitis and DED. This review aims to provide theoretical foundations for the potential application of natural products in the treatment of ocular inflammatory diseases.

### 1.1. Inflammation in Ocular Diseases

The eye is an immune-privileged organ, and this privilege is essential for maintaining ocular homeostasis [18]. However, when immune tolerance is disrupted, immune activation can readily trigger excessive and persistent inflammatory responses, with effector T cells recognizing ocular antigens and initiating inflammatory cascades, leading to chronic ocular inflammation [12,19].

Both innate and adaptive immune responses are involved in ocular inflammatory diseases [20,21]. In particular, Th17 cells serve as critical drivers by secreting IL-17 and other pro-inflammatory cytokines, which are considered key contributors to the early pathogenic processes [10,11]. Regulatory T cells (Tregs) are responsible for maintaining immune homeostasis and suppressing excessive inflammatory responses through the secretion of anti-inflammatory cytokines, such as interleukin-10 (IL-10) and transforming growth factor-β (TGF-β) [22,23]. However, in ocular disease patients, a pronounced increase in Th17 cells is accompanied by a reduction in both the abundance and regulatory function of Tregs [24,25,26]. This Th17/Treg imbalance leads to sustained activation of inflammatory cascades and exacerbates disease progression.

In addition, when the ocular surface sustains injury or is exposed to stress, innate immune responses are activated [15]. This process is accompanied by elevated NLRP3 inflammasome activation, which promotes increased release of downstream pro-inflammatory cytokines, such as IL-1β and IL-18 [27]. These cytokines further recruit peripheral T cells to the site of inflammation and contribute to tissue damage and immunopathological processes, thereby reinforcing chronic inflammatory responses [10,28].

**Figure 1:**
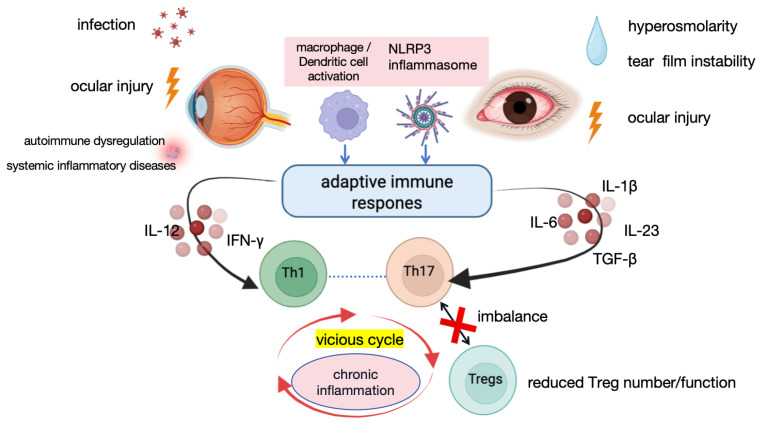
Mechanisms of Uveitis and DED. Dysregulated adaptive immune responses underlie chronic ocular inflammation [6,8,12,20,21,24]. Various stimuli, including infection, ocular injury, autoimmune dysregulation, systemic inflammatory diseases, and tear film hyperosmolarity, activate macrophages and dendritic cells and trigger NLRP3 inflammasome activation. These innate immune events promote the release of pro-inflammatory cytokines, such as IL-12, IL-6, IL-1β, IL-23, and TGF-β, thereby inducing Th1 and Th17 differentiation. Imbalanced Th1/Th17 responses, together with reduced numbers and/or impaired suppressive function of Tregs, disrupt immune homeostasis, forming a self-amplifying inflammatory cycle that ultimately leads to persistent inflammation and ocular tissue damage. Abbreviations: NLRP3, NOD-like receptor family pyrin domain containing 3; IL, interleukin; TGF-β, transforming growth factor-β; Tregs, regulatory T cells; Th17, T helper 17; Th1, T helper 1 cells; IFN-γ, interferon-γ. Figure created in BioRender. Q.L. (2026) https://BioRender.com/vr5o8so (accessed on 29 March 2026).

This immune dysregulation-driven inflammation forms the immunopathological basis of several representative ocular inflammatory disorders, including uveitis and DED, as shown in Table 1 and Figure 1.

### 1.2. Inflammation in Uveitis

Uveitis is generally classified into infectious and noninfectious types. Despite its highly heterogeneous etiologies, accumulating evidence indicates that abnormal activation of the immune system plays a key role in pathogenesis and progression, regardless of the underlying cause [21].

Noninfectious uveitis encompasses a spectrum of immune-mediated intraocular inflammatory disorders, in which adaptive immune dysregulation is accompanied by innate immune amplification, particularly involving macrophages and inflammasome signaling, which further amplifies local inflammatory cascades and creating a self-sustaining inflammatory network [29]. Activated immune pathways (including NF-κB/MAPK-related signaling axes) promote cytokine and chemokine production, leukocyte recruitment [30], and blood–ocular barrier disruption, all of which contribute to recurrent inflammation and structural damage. Accumulating evidence indicates that aberrant activation of adaptive immunity, particularly excessive Th1 and Th17 responses, plays a central role in initiating and sustaining intraocular inflammation [19]. Meanwhile, impaired Treg number and/or function weakens anti-inflammatory counter-regulation, further supporting chronic disease activity [31,32].

This imbalance between pro-inflammatory effector responses and regulatory mechanisms establishes a permissive microenvironment for sustained immune activation, which may predispose patients to recurrent disease and incomplete immune resolution even after apparent clinical improvement.

Collectively, these mechanisms help explain why long-term remission is difficult in a subset of patients despite short-term inflammatory control. In clinical practice, uveitis is typically managed using etiology-based therapy (e.g., anti-infective treatment for infectious uveitis), together with corticosteroids and/or steroid-sparing immunomodulatory treatment when indicated [33,34]. However, prolonged treatment is frequently associated with ocular complications such as cataract and glaucoma, as well as systemic adverse effects (e.g., cardiovascular events and hepatotoxicity), and treatment cost can impose a substantial financial burden [2,35].

Among infectious uveitis entities, cytomegalovirus-associated anterior uveitis (CMV-AU) has attracted increasing attention in recent years and appears to be particularly prevalent in Asian populations.

CMV-AU is discussed here as an infectious uveitis exemplar because it combines antiviral treatment dependency with persistent immune-mediated inflammation, making it a useful model for discussing adjunctive immunomodulatory strategies. This condition was historically recognized predominantly in immunocompromised individuals; however, emerging evidence indicates that CMV-AU can also develop in immunocompetent patients [3]. These findings suggest that while CMV infection serves as the initiating trigger in CMV-AU, accumulating evidence indicates that the persistence and recurrence of inflammation are substantially influenced by host immune responses [3]. Indeed, the interplay between viral replication and immune-mediated pathology appears to determine the clinical course of the disease. Furthermore, CMV infection may contribute to persistent ocular inflammation in both immunocompromised and immunocompetent individuals, although mechanisms likely differ.

Research indicates that CMV infection not only directly affects ocular tissues but also profoundly alters the functional phenotype of host immune cells [36]. During infection, CD4^+^ T cells differentiate into distinct subsets, including pro-inflammatory Th1/Th17 cells and anti-inflammatory Tregs [37,38,39], contributing to a chronic inflammatory state in CMV-AU. Notably, an increased Th17/Treg ratio has been reported in individuals with human cytomegalovirus (HCMV) infection, showing similarities to the immune imbalance observed in EAU [40,41]. Evidence also suggests that the higher prevalence of CMV-AU in males may be associated with stronger CD4^+^ T cell responses [42].

Moreover, a pro-inflammatory cytokine milieu may facilitate CMV reactivation in some settings, potentially contributing to recurrent inflammation [43]. As a consequence, many CMV-AU patients experience recurrent inflammation after treatment discontinuation, suggesting that antiviral therapy alone (e.g., ganciclovir/valganciclovir) may not always prevent recurrence and that adjunctive inflammation control may be required in selected patients [44,45].

Overall, noninfectious uveitis and CMV-AU differ in initiating triggers but share downstream inflammatory nodes (e.g., cytokine amplification and Th17/Treg disequilibrium). This convergence supports investigation of multi-target immunomodulatory approaches, including natural products discussed in the following sections. However, it should be noted that the initiating triggers differ substantially between infectious and noninfectious uveitis. Therefore, extrapolation of mechanisms across these disease settings should be interpreted with caution, particularly for disease initiation and pathogen-driven pathology.

### 1.3. Inflammation in DED

DED is one of the most prevalent ocular surface diseases worldwide. Its prevalence increases significantly with age, leading to substantial impairment of individuals’ quality of life and imposing a heavy economic burden [46]. With the ongoing global aging trend, the number of people is projected to reach approximately 2.1 billion by 2050, suggesting that the prevalence of DED is likely to rise further in the coming decades [47].

Common risk factors of DED include vitamin A deficiency, improper contact lens wear, and use of ophthalmic products containing preservatives such as benzalkonium chloride [48,49]. The central symptoms are characterized by dryness, grittiness, foreign body perception, photophobia, burning sensation, and vision fluctuation, accompanied by chronic ocular surface inflammation that further compromises ocular surface integrity and visual function [50].

Conventional clinical management of DED primarily includes artificial tear supplementation, punctal plug insertion, and physical therapy for the meibomian glands [51]. These symptomatic treatments are effective in alleviating ocular discomfort in the mild DED patients, as for moderate to severe cases, first-line anti-inflammatory treatments mainly include glucocorticoids and cyclosporine A. Although these agents show anti-inflammatory efficacy, prolonged corticosteroid use is associated with adverse ocular effects such as glaucoma, cataract, and increased infection risk [13,52]. However, due to its pharmacokinetic limitations, cyclosporine A is difficult to deliver at effective concentrations in the cornea and may induce ocular burning sensations. Although the newly approved 0.09% cyclosporine A ophthalmic nanomicellar solution in 2018 exhibits better solubility and bioavailability, high-quality evidence in severe DED remains limited [53,54].

With the advancement of research in recent years, therapeutic strategies for DED have gradually shifted toward anti-inflammatory therapy. Lifitegrast, an integrin antagonist approved in 2016, was among the first targeted therapies for DED specifically designed to inhibit T cell-mediated inflammatory signaling pathways. Clinical trials have demonstrated that lifitegrast significantly alleviates DED symptoms [55], suggesting that agents capable of modulating inflammatory reactions and immune-related pathways may have potential advantages in the management of DED.

**Table 1:** Representative immunological readouts across ocular inflammatory disease contexts.

Key Readout	Typical Assay	DED (Sample/Interpretation)	Noninfectious Uveitis (Sample/Interpretation)	CMV-AU (Sample/Interpretation)	Practical Note	References
IL-17	Western blot/qPCR	Conjunctival/corneal tissue (assay-dependent); hallmark of Th17-driven ocular surface inflammation.	EAU ocular tissue; key effector cytokine in inflammatory cascade amplification.	Aqueous humor/ocular inflammatory samples (assay-dependent); reflects secondary Th17 activation during persistent immune inflammation.	Interpret as a local ocular inflammatory readout and alongside other cytokines/viral burden.	[8,10,12]
IFN-γ	Western blot/qPCR	Conjunctival/corneal tissue; associated with Th1 activation and chronic inflammation.	Aqueous/vitreous samples; associated with Th1 activation and chronic intraocular inflammation.	Aqueous humor; key antiviral cytokine reflecting Th1-driven activation and macrophage stimulation.	Interpret with other cytokines to avoid overcalling Th1 dominance.	[12,15,43]
IL-6	ELISA/qPCR	Tear samples and/or conjunctival-corneal tissue (assay-dependent); promotes Th17 differentiation and amplifies ocular surface inflammation.	Aqueous/vitreous samples; promotes Th17 differentiation and amplifies intraocular inflammatory cascades.	Aqueous humor; associated with active viral infection and inflammatory activation.	Useful as an adjunct inflammatory marker, not a stand-alone diagnostic marker.	[8,12,25]
Th17/Treg ratio	Flow cytometry	Conjunctival immune-cell samples (or study-specific ocular samples); indicates immune imbalance linked to severity/chronicity.	Aqueous/vitreous cellular samples (study-dependent); indicates immune imbalance linked to severity/chronicity.	Aqueous humor cellular fraction (study-dependent); may reflect persistent immune dysregulation during/after viral control.	Method-dependent metric; gating strategy and sampling approach should be standardized.	[9,12,24,41]
FoxP3	Flow cytometry	Conjunctival immune-cell samples (or study-specific ocular samples); marker of Treg identity/function and immune tolerance.	Aqueous/vitreous cellular samples (study-dependent); marker of Treg identity/function and immune tolerance.	Aqueous humor cellular fraction (study-dependent); marker of Treg identity/function and immune tolerance.	Reflects Treg identity/function rather than absolute Treg count.	[22,31]
NLRP3	Western blot/qPCR	Conjunctival/corneal tissue; supports inflammasome involvement in ocular surface inflammation.	Aqueous/vitreous samples; promotes downstream pro-inflammatory cytokine expression and immune amplification.	Aqueous humor; may reflect inflammasome-linked immune activation in CMV-AU.	Consider with IL-1β/IL-18 when available.	[27,56]
MMP-9	Tear ELISA	Tear fluid; reflects ocular surface epithelial damage and barrier dysfunction in DED.	-	-	Widely used clinical biomarker of ocular surface inflammation in DED.	[15]
CMV DNA	Quantitative PCR	-	-	Aqueous humor; diagnostic reference standard for suspected CMV-AU.	Aqueous humor PCR serves as the key specimen for detecting CMV DNA and distinguishing CMV-AU from noninfectious immune-mediated uveitis.	[3]

Abbreviations: DED, dry eye disease; CMV-AU, cytomegalovirus-associated anterior uveitis; EAU, experimental autoimmune uveitis; qPCR, quantitative polymerase chain reaction; Th17, T helper 17; Treg, regulatory T cells; IL-1β, interleukin-1β; IL-18, interleukin-18; IL-17, interleukin-17; IFN-γ, interferon-γ; Th1, T helper 1 cells; ELISA, enzyme-linked immunosorbent assay; IL-6, interleukin-6; FoxP3, forkhead box P3; NLRP3, NOD-like receptor family pyrin domain containing 3; MMP-9, matrix metalloproteinase-9.

Collectively, these findings indicate that ocular inflammatory diseases are sustained by interconnected immune pathways rather than a single mediator. Therefore, in the following sections, we evaluate representative natural products according to their effects on shared inflammatory nodes—particularly Th17/Treg balance, NF-κB/MAPK-associated signaling, inflammasome activity, and oxidative stress—and discuss their translational relevance to uveitis and DED.

### 1.4. Scope and Literature Search Strategy

This narrative review focuses on natural products with reported immunomodulatory potential in ocular inflammatory diseases, with particular emphasis on uveitis, noninfectious uveitis, CMV-AU, and DED. The literature was searched in PubMed, Web of Science, and Google Scholar for articles published in English up to March 2026. Search terms included combinations of “uveitis”, “dry eye disease”, “ocular inflammation”, “ocular immune mechanisms”, “cytomegalovirus-associated anterior uveitis”, “natural products”, “Paeonia lactiflora”, “paeoniflorin”, “resveratrol”, “pterostilbene”, “polydatin”, “curcumin”, “boswellic acids”, “immunomodulation”, “NF-κB”, “MAPK”, “NLRP3 inflammasome”, and “Th17/Treg”. Priority was given to original studies and clinically relevant reports addressing immunological mechanisms, ocular delivery, and translational potential in ocular inflammatory diseases. Additional relevant articles were identified through manual screening of the reference lists of selected papers. As this is a narrative review, the literature was selected to provide mechanistic breadth and translational relevance rather than a formal systematic synthesis.

## 2. Natural Products as Immunomodulatory Therapeutic Strategies

Ocular tissues exhibit a relatively low tolerance to inflammation, and mild inflammation can impair the visual function, even leading to irreversible blindness. Therefore, excessive or persistent chronic inflammation requires timely and effective control [57].

To date, there is no universally recognized therapeutic strategy that effectively controls inflammation driven by immune dysregulation, and current clinical management strategies for uveitis or DED remain limited, especially in meeting the long-term and stable therapeutic needs of moderate to severe patients.

Against this background, natural product-derived agents have attracted increasing attention for their prevention and management of various chronic diseases, due to their low cytotoxicity, favorable safety profiles, and accessible sources [58].

Compared with conventional synthetic drugs, natural products are characterized by diverse chemical structures, broad-spectrum action pathways and relatively lower toxicity. By acting on multiple molecular targets, natural products coordinately modulate inflammatory responses, oxidative stress and immune cell function, thereby exhibiting unique advantages in complex inflammatory diseases. In recent years, a growing body of research has demonstrated that natural products possess potential capacity to restore immune homeostasis, suppress pro-inflammatory cytokines secretion, and protect tissue integrity in various immune associated diseases models, supporting the new insights into the development of long-term intervention in chronic ocular disease [16].

**Figure 2:**
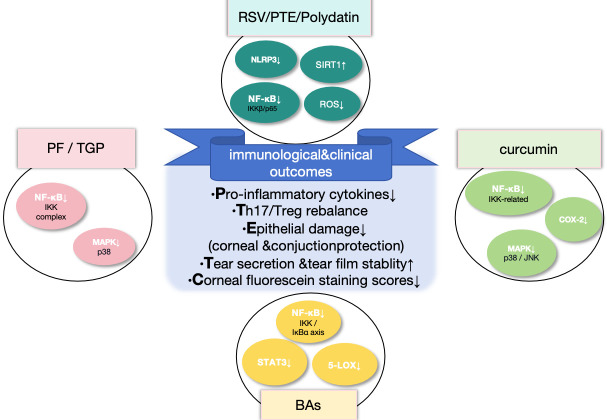
Immunomodulatory and clinical effects of representative natural compounds. RSV/PTE/Polydatin [70,96], curcumin [81,97,98], BAs [58,99], and PF/TGP [100,101] exert convergent anti-inflammatory and immunoregulatory effects by targeting key signaling pathways, including NF-κB, MAPK/p38, STAT3, NLRP3 inflammasome, COX-2, 5-LOX, SIRT1, and ROS. Upward and downward arrows indicate upregulation and downregulation, respectively. Compounds sharing similar mechanisms are grouped; references correspond to representative studies only. These interventions suppress pro-inflammatory cytokine production, restore Th17/Treg balance, and attenuate epithelial inflammatory injury, thereby protecting the corneal and conjunctival tissues. At the clinical level, they improve tear secretion and tear film stability and reduce corneal fluorescein staining scores, indicating improved ocular surface integrity. Abbreviations: RSV, resveratrol; PTE, pterostilbene; BAs, boswellic acids; PF, paeoniflorin; TGP, total glucosides of peony; ROS, reactive oxygen species; NLRP3, NOD-like receptor family pyrin domain containing 3; SIRT1, sirtuin 1; NF-κB, nuclear factor kappa B; STAT3, signal transducer and activator of transcription 3; IKK, IκB kinase; IκBα, inhibitor of κB alpha; MAPK, mitogen-activated protein kinase; COX-2,cyclooxygenase-2; 5-LOX,5-lipoxygenase; JNK, c-Jun N-terminal kinase. Figure created by the authors.

Based on these considerations, the following sections will focus on several representative natural products, including *Paeonia lactiflora*, resveratrol, curcumin, and boswellic acids, and systematically summarize their anti-inflammatory and immunoregulatory mechanisms as shown in Table 2 and Figure 2. Furthermore, their potential research value and future prospects as novel therapeutic strategies for uveitis and DED will be discussed in the context of current experimental and clinical evidence.

### 2.1. Paeonia lactiflora

*Paeonia lactiflora* is a traditional medicinal herb widely used in Asia and has been extensively investigated for its therapeutic potential in recent years. Studies have reported that various extracts of *Paeonia lactiflora* exhibited multiple effects, including antioxidant, anti-inflammatory, and immunomodulatory properties. These extracts have been clinically applied in China and Japan for the treatments of immune-related disorders such as rheumatoid arthritis (RA), systemic lupus erythematosus, and hepatitis, and have been reported to possess favorable safety profile at effective concentrations [59].

The water and ethanol extracts of *Paeonia lactiflora*, known as total glucosides of peony (TGP), contain paeoniflorin as the predominant bioactive constituent, which accounts for more than 40% of the total composition [60]. Substantial experimental evidence has shown that TGP possesses pronounced anti-inflammatory and immunomodulatory activities. These effects are mediated through modulation of the NF-κB signaling pathway and regulation of macrophage polarization, thereby restoring the M1/M2 balance [61]. NF-κB signaling is widely recognized as one of the central molecular targets mediating the biological effects of TGP [60]. Moreover, TGP has been shown to reduce the expression of downstream pro-inflammatory cytokines in systemic autoimmune models including TNF-α, IL-1β, IL-17, and IFN-γ, thereby contributing to the attenuation of inflammatory cascade amplification [62,63].

Furthermore, TGP has been reported to contribute to reshaping the immune microenvironment by regulating macrophage subsets polarization and dendritic cell migration/function, inhibiting excessive proliferation of Th1 and Th17 cells [59,60]. Overall, these multiple-layered immunomodulatory effects may provide an important theoretical basis for the application of *Paeonia lactiflora* extracts in chronic inflammatory and immune-related diseases.

Recent studies also have explored the effect of these bioactive components in ocular inflammation, providing supportive experimental evidence. Liu et al. used NOD mouse model of Sjögren’s syndrome to systematically evaluate the immunomodulatory effects of TGP. Results showed that TGP significantly reduced the Th1/Th17 ratio in peripheral blood, accompanied by decreased expression of pro-inflammatory mediators such as IL-17A, IFN-γ, and IL-2. In addition, TGP could suppress Th1/Th17 related transcriptional activity, including the expression of T-bet. Functionally, treatment with TGP increased tear and saliva secretion compared with the vehicle control group, suggesting a therapeutic benefit in alleviating glandular secretory function [64].

Moreover, through in vitro and in vivo experiments, Zhao et al. investigated the effects of paeoniflorin (PF) by using human corneal epithelial cells and mouse model of dry eye. Under hyperosmolar stress, PF has been reported to reduce the mRNA expression of pro-inflammatory cytokines in human corneal epithelial cells in a dose-dependent manner. In the mouse dry eye model, PF treatment enhanced tear secretion as well as reduced corneal fluorescein staining scores, suggesting its capacity to preserve corneal epithelial integrity and alleviate ocular surface damage [65].

Taken together, these experimental findings indicate that *Paeonia lactiflora* extracts exhibit distinct biological effects by modulating Th1/Th17-related immune responses, suppressing pro-inflammatory cytokine expression, and improving both tear film and corneal epithelial integrity. This supports further investigation and potential adjunctive approach of *Paeonia lactiflora* extracts in chronic ocular surface diseases.

However, traditional TGP exhibits low absorption and bioavailability following oral administration due to its susceptibility to extensive metabolism by the gut microbiota [59], which may limit its clinical application. Recent research has shown that *Paeonia lactiflora* extracts offer certain pharmacological advantages, not only displaying enhanced antioxidant and anti-inflammatory effects, but are also enriched in other bioactive compounds, particularly pyrogallol. Existing studies indicates that pyrogallol can significantly attenuate inflammatory reactions and regulate the expression of multiple pro-inflammatory genes, thereby contributing to immunomodulatory and potentially enhancing the therapeutic efficacy of *Paeonia lactiflora* extracts [66]. Consequently, these findings suggest that optimizing the preparation process, such as through fermentation may enhance its bioavailability and strengthen anti-inflammatory and immunomodulatory effects, support further investigation into the application of *Paeonia lactiflora* derived natural products in chronic inflammatory diseases.

### 2.2. Resveratrol

Polyphenols are considered one of the most prominent bioactive constituents among the natural products, and many therapeutic applications and formulations have been explored for polyphenolic compounds [67]. Polyphenols exhibit broad pharmacological activities across cardiovascular diseases, cancer, neurological disorders, aging, and inflammatory conditions [68]. Among these, resveratrol (RSV) is the most extensively studied and has been widely investigated because of its broad-spectrum anti-inflammatory and neuroprotective properties in both experimental and diseases-related studies.

RSV is widely distributed in nature and occurs in grapes, soybeans, peanuts, and certain plants such as eucalyptus. Early study suggested that polyphenolic compounds present in red wine showed cardiovascular protective effects, prompting extensive investigation into RSV as an anti-inflammatory and antioxidant properties [69].

RSV exerts anti-inflammation effects by activating the Sirtuin-1 (SIRT1) signaling pathway, thereby suppressing the expression of multiple pro-inflammatory cytokines, including IL-17, IL-6, TNF-α, and IL-23. Additionally, RSV has been reported to adjust immune response by interfering with immune-cell functions, suppress the synthesis of pro-inflammatory cytokines, and inhibit the expression of their associate genes [70]. Research has shown that RSV participated in regulating the activation of macrophages [71], effector T cells, and natural killer (NK) cells, while also influencing the immunosuppressive function of CD4^+^CD25^+^ regulatory T cells.

Its anti-inflammatory effects are closely associated with the neutralization of reactive oxygen species (ROS), inhibition of cyclooxygenase activity (COX-1 and COX-2) [69], and activation of multiple anti-inflammatory signaling pathways. Moreover, RSV exerts antioxidant and anti-inflammatory activities [72], suggesting potential therapeutic value in inflammation-associated ocular diseases.

Shunsuke Kubota et al. reported in an endotoxin-induced uveitis mouse model that oral resveratrol supplementation attenuated oxidative stress-related damage and suppressed the activation of the NF-κB signaling pathway, thereby exerting anti-inflammatory effects and contributing to reduced intraocular inflammation [72].

Similarly, in a mouse model of DED, RSV treatment has been shown to reduce the levels of multiple inflammatory cytokines in tear fluid of treated mice, suggesting a potential benefit in the management of ocular surface inflammation [71].

Despite extensive research supporting that RSV possesses favorable safety profiles and well-established anti-inflammatory and antioxidant effects [73], its translation into clinical practice remains limited. Due to its low oral bioavailability, rapid metabolism and poor aqueous solubility, RSV may fail to maintain stable and effective concentrations in target tissues, which could constrain its development as a therapeutic agent [74]. Currently, RSV is more commonly used as a dietary supplement or as an adjunctive intervention.

In recent years, increasing attention has shifted toward resveratrol analogs and precursor compounds. Among these, pterostilbene (PTE; trans-3,5-dimethoxy-4′-hydroxystilbene) and polydatin, a glucoside derivative of resveratrol, are the two most extensively investigated compounds. PTE is a dimethylated analog of resveratrol, primarily derived from red sandalwood. Compared with RSV, PTE has been reported to exhibit better lipophilicity, resulting in higher intestinal absorption, bioavailability and biological activity [75]. Beyond sharing the well-recognized anticancer, anti-inflammatory, and antioxidant properties of resveratrol derivatives, PTE has also been reported to possess unique antiviral effects. Wang et al. were the first to investigate the inhibitory effects and underlying regulatory mechanisms of PTE against HCMV infection through an in vitro WI-38 cell model. Their results suggested that PTE preserved the morphological characteristics of infected cells and markedly suppressed HCMV DNA replication [76]. In summary, PTE inhibited viral infection at multiple levels, including protein expression, gene transcription, oxidative stress status, and viral titers, further supporting its potential antiviral activity against HCMV under in vitro conditions and warranting further investigation.

Polydatin is mainly derived from *Polygonum cuspidatum*, a Japanese traditional medicinal herb. Similarly to RSV, polydatin exhibits pronounced antioxidant and anti-inflammatory activities while it has superior aqueous solubility and chemical stability. Polydatin has been observed to modulate key inflammatory signaling pathways, including NF-κB and the NLRP3 inflammasome, inhibiting the expression of downstream pro-inflammatory cytokines [77,78]. Its therapeutic potential has been reported in cardiovascular and oncologic contexts, while robust clinical evidence in ocular diseases remains limited.

### 2.3. Curcumin

Curcumin is a natural polyphenol extracted from the rhizomes of Curcuma species, chemically defined as 1,7-bis(4-hydroxy-3-methoxyphenyl)-1,6-heptadiene-3,5-dione. It exhibits diverse pharmacological activities including antioxidant, anti-apoptotic, and anti-inflammatory, gaining considerable attention due to its favorable safety profile and minimal adverse reactions [79].

Research indicated that, compared to conventional single-target anti-inflammatory agents, curcumin exhibits multiple-target modulatory properties. It suppresses classical inflammatory signaling pathways, including p38 MAPK and NF-κB, and also reduces the activity of cyclooxygenase-2 (COX-2), thereby contributing to coordinated anti-inflammatory effects. Through these mechanisms, curcumin has been associated with downregulated expression of pro-inflammatory cytokines, including IL-1β, IL-6, IL-12, IFN-γ, and TNF-α, while upregulating anti-inflammatory cytokines such as IL-4 and IL-10. In addition, curcumin has been reported to influence the Th17/Treg balance, thereby promoting the restoration of immune homeostasis and attenuating inflammation and tissue infiltration. Moreover, curcumin possesses high antioxidant properties and acts as a natural scavenger of reactive oxygen species (ROS), it may help mitigate the amplification of inflammatory pathways, suggesting a potential modulatory role of curcumin in various inflammatory disease [80,81,82,83].

Clinical research has demonstrated that curcumin may exhibit therapeutic efficacy in asthma [84], psoriasis, arthritis, and ulcerative colitis [85]. These benefits are associated with reduced serum levels of pro-inflammatory cytokines, enhanced expression of anti-inflammatory cytokines, modulation of the Th17/Treg balance, and attenuation of inflammatory responses and disease progression [81].

Curcumin also showed its therapeutic potential for ocular disease. Oral administration of curcumin has been reported to show beneficial effects in chronic anterior uveitis and reduction in disease recurrence [86]. Liu et al. demonstrated in a benzalkonium chloride induced dry eye mouse model that oral administration of curcumin reduced the expression of pro-inflammatory cytokines, modulated oxidative stress–related pathways, accelerated corneal epithelial repair, ultimately alleviating apoptosis and inflammatory responses [87]. In a randomized, double-blind, placebo-controlled trial involving 40 DED patients, curcumin treatment improved ocular surface parameters, including visual acuity, tear secretion, corneal staining, TBUT, and OSDI score, compared with placebo [88]. Another study on oxidative stress in human corneal epithelial cells indicated that, compared with the control group, curcumin treatment reduced NF-κB expression, accompanied by downregulation of the inflammasome protein NLRP3 and pro-inflammatory cytokines such as TNF-α [89]. These findings suggested that curcumin may modulate oxidative stress-induced inflammation and may exert effects on corneal epithelial cells in vitro, supporting further investigation into the potential application of curcumin-based formulations in ophthalmic diseases.

Collectively, accumulating evidence suggests that curcumin, as a naturally derived multi-target anti-inflammatory agent, may modulate inflammatory cascades reactions. It has shown potential in ocular inflammatory diseases with advantages in clinical applications such as a favorable safety profile, good tolerability, and cost-effectiveness [82,86].

However, some limitations of polyphenols such as poor oral absorption, rapid metabolism, and low bioavailability may hinder clinical translation. In recent years, structural modifications for curcumin, including the development of derivatives and analogs, as well as advanced delivery systems such as hydrogels, nanoparticles, and liposomal formulations [89], have been explored as potential strategies to improve pharmacokinetic drawbacks. Meanwhile, combination therapy approaches have attracted increasing attention. Co-administration with agents such as boswellic acids, vitamin D, or piperine has been reported to enhance the bioavailability of curcumin and offers synergistic anti-inflammatory or immunomodulatory effects in multiple research [86]. Presently, dietary supplement formulations remain one of the most practical forms of curcumin use. However, its therapeutic efficacy still requires validation through larger-scale clinical data [81,82].

### 2.4. Boswellic Acids

Boswellic acids (BAs) are a class of pentacyclic triterpenoids derived from the resin of Boswellia species, among which *Boswellia serrata* is the main medicinal source. To date, acknowledged primary bioactive constituents include 3-O-acetyl-11-keto-β-boswellic acid (AKBA), 11-keto-β-boswellic acid (KBA), and β-boswellic acid (BA). Substantial evidence indicates that BAs exert pronounced anticancer, antiviral, and anti-inflammatory effects by regulating the NF-κB signaling pathway and apoptosis-associated genes expression. BAs also broadly participate in the regulation of oxidative stress and inflammation, demonstrating considerable therapeutic potential in tumor initiation and progression [90].

In recent years, driven by the pronounced anti-inflammatory potential of BAs, numerous preclinical and clinical research have advanced rapidly. Mechanistically, BAs have been reported to modulate several inflammation-related signaling pathways [91]. By targeting key inflammation mediators, such as NF-κB, STAT3, cyclooxygenase-2 (COX-2), 5-lipoxygenase (5-LOX), and matrix metalloproteinase-9 (MMP-9) [58]. Research has shown that BAs are associated with reduced expression of downstream pro-inflammatory cytokines, including IL-6, IL-1β, and TNF-α, thereby attenuating inflammatory responses and limiting bone tissue destruction [92].

In addition, BAs also have been reported to regulate immune cell differentiation, including suppression of M1 macrophage polarization and modulation of Th1/Th17 balance, thereby promoting the immune homeostasis and attenuation of inflammation-induced tissue damage [93]. Regarding immunomodulation effect, Meyiahabdo et al. demonstrated that BAs enhance Treg functional activity in vitro, with upregulation of FoxP3 and Helios despite no change in Treg proportion [94].

The anti-inflammation and immunomodulation of BAs have also been supported by in vivo studies. Singh et al. demonstrated that topical formulations of BAs were associated with attenuation of ear and paw swelling and arthritis-related manifestations in a dose-dependent manner [91]. In addition, Chen LungChe et al. observed that oral pretreatment with BAs was associated with reduced hepatic inflammatory cell infiltration, Ishak scores, and the expression of key inflammatory proteins such as NF-κB and p65, suggesting potential suppression of inflammatory signaling pathways [95].

Due to the reported anti-inflammatory properties of BAs, preclinical and clinical research have advanced rapidly. Combination therapy with BAs and curcumin may represent a potential clinical strategy. A clinical study involving 100 individuals with chronic back pain demonstrated that CL0192, a formulation containing boswellic acids and curcumin, reduced serum levels of pro-inflammatory cytokines and improved clinical symptoms in the treatment group. The formulation was well tolerated, with no apparent side effects observed. BAs are suggested to exert anti-inflammatory effects that may act synergistically with curcumin [80].

Although accumulating evidence from studies supports the therapeutic potential and favorable safety of BAs in various inflammatory diseases, evidence in ocular diseases remains limited. Moreover, some pharmacokinetic reports suggest short systemic exposure, potentially requiring frequent dosing [58]. Therefore, further large-scale, well-designed studies are needed to evaluate BAs’ therapeutic value and clinical feasibility in ocular inflammation.

**Table 2:** Mechanisms and experimental evidence of natural products in ocular inflammatory diseases.

Natural Product	Major Targets	Key Immunological Effects	Representative Ocular Evidence/Disease Context	Evidence Level	Administration Route Tested	Main Limitations	Key References
Total glucosides of peony (TGP)	NF-κB signaling pathway	Reduced pro-inflammatory cytokines; modulation of macrophage subset imbalance	NOD mouse model of Sjögren’s syndrome-related ocular surface inflammation	Animal study	Oral gavage	Moderate bioavailability; intestinal metabolism	[64]
Paeoniflorin (PF)	NF-κB signaling pathway; oxidative stress-related pathways	Reduced pro-inflammatory cytokines; attenuation of epithelial inflammatory injury; antioxidative effects	Human corneal epithelial cells under hyperosmolar stress; mouse model of DED	Cell and animal studies	In vitro cell treatment; topical ocular administration	Limited ocular pharmacokinetic data; bioavailability concerns	[65]
Resveratrol (RSV)	SIRT1 signaling pathway; NF-κB-related inflammatory signaling	Reduced pro-inflammatory cytokines; regulation of immune cell function; reduced oxidative stress-linked inflammation	Mouse model of endotoxin-induced uveitis; mouse model of DED	Animal studies	Oral administration; topical ocular administration	Extensive first-pass metabolism; low systemic bioavailability; poor water solubility	[71]
Polydatin	NF-κB signaling pathway; NLRP3 inflammasome	Reduced pro-inflammatory cytokines; suppression of inflammasome-related inflammation; epithelial protection	Rat model of exorbital lacrimal gland excision-induced dry eye; human conjunctival epithelial cell system	Animal and cell studies	Topical ocular administration; in vitro cell treatment	Limited ophthalmic translational evidence	[78]
Pterostilbene (PTE)	SIRT1-related signaling; antiviral and anti-inflammatory pathways	Anti-inflammatory effects; potential antiviral activity; reduced oxidative stress-linked inflammation	In vitro studies relevant to CMV/HCMV-associated inflammatory conditions; no direct ophthalmic clinical study	Cell study only; no ophthalmic clinical evidence	In vitro cell treatment	No direct ophthalmic clinical evidence; limited ocular delivery data	[76]
Curcumin	p38 MAPK and NF-κB signaling pathways	Reduced pro-inflammatory cytokines; increased anti-inflammatory cytokines; modulation of Th17/Treg balance; reduced oxidative stress-linked inflammation	Human anterior uveitis cohort; mouse model of DED; human DED randomized controlled trial; human corneal epithelial cells	Clinical studies (including RCTs), animal and cell studies	Oral administration; in vitro cell treatment	Extremely poor solubility; rapid metabolism; chemical instability	[86,87,88,89]
Boswellic acids (BAs)	NF-κB and STAT3 signaling pathways	Reduced pro-inflammatory cytokines; reduced M1 macrophage polarization; reduced Th1/Th17 responses; enhanced Treg-associated regulation	No direct ophthalmic clinical study; mainly indirect or non-ocular preclinical evidence with possible ocular relevance	Preclinical evidence only; no ophthalmic clinical evidence	Mainly oral administration in non-ocular studies	Poor aqueous solubility; limited oral absorption; rapid metabolism; lack of ophthalmic evidence	[80,93]

Abbreviations: TGP, total glucosides of peony; PF, paeoniflorin; RSV, resveratrol; PTE, pterostilbene; BAs, boswellic acids; NF-κB, nuclear factor kappa B; NLRP3, NOD-like receptor family pyrin domain containing 3; STAT3, signal transducer and activator of transcription 3; SIRT1, sirtuin 1; MAPK, mitogen-activated protein kinase; Th17, T helper 17; Treg, regulatory T cells; NOD, non-obese diabetic; CMV, cytomegalovirus; HCMV, human cytomegalovirus; RCTs, randomized controlled trials; M1, classically activated macrophages; Th1, T helper 1.

### 2.5. Integrative Pathogenic Framework and Therapeutic Convergence

Taken together, although the upstream triggers differ across DED, noninfectious uveitis, and CMV-AU, the reviewed natural products appear to converge on a limited set of interacting pathogenic modules: (1) innate immune activation, including NF-κB/MAPK and NLRP3 inflammasome signaling; (2) adaptive immune disequilibrium, particularly Th17/Treg imbalance; (3) cytokine and oxidative-stress amplification; and (4) epithelial or tissue injury that perpetuates chronic inflammation. This framework may help explain why multi-target natural compounds can show overlapping anti-inflammatory effects across distinct ocular disease settings despite differences in disease initiation.

## 3. Comparative Evaluation and Challenges of Natural Products in Ophthalmic Applications

From a translational perspective, the four categories of natural products discussed in this review appear to be at different stages of clinical maturity. Among them, curcumin currently seems to be the most advanced candidate for ocular application because it is supported by both preclinical ocular studies and early human evidence, including a clinical study in anterior uveitis and a randomized controlled trial in dry eye disease. In comparison, RSV and its derivatives offer broad mechanistic versatility, including anti-inflammatory, antioxidant, and context-dependent antiviral activities, and may therefore be of particular interest, whereas the other three categories primarily act as adjunctive modulators of downstream inflammatory amplification rather than direct antiviral agents. However, their ophthalmic evidence is still largely preclinical, and their pharmacokinetic limitations continue to restrict direct translation. *Paeonia lactiflora* derivatives, particularly TGP and PF, show promising immunomodulatory effects in ocular surface inflammation and autoimmune-like settings, but current ocular evidence remains mainly experimental, indicating an earlier phase of clinical translation. BAs have compelling anti-inflammatory and immune-regulatory properties, but the current ophthalmic evidence is the least mature and remains largely indirect or preclinical. Overall, curcumin appears closest to short-term clinical application, whereas RSV and its derivatives may hold strong medium-term translational potential. In contrast, *Paeonia lactiflora* derivatives and BAs remain promising but earlier-stage candidates require additional ocular and clinical validation.

However, their translation into ophthalmology remains limited by several challenges. First, the unique anatomical and physiological barriers of the eye significantly restrict drug delivery efficiency. Rapid tear turnover, blinking, and nasolacrimal drainage lead to rapid drug clearance, with ocular bioavailability typically below 5% [102]. In addition, the tight junctions of the corneal epithelium and the vascularized conjunctiva limit intraocular penetration while facilitating systemic absorption, thereby reducing local drug concentrations [103]. These factors often necessitate frequent dosing, which may affect patient compliance. Furthermore, the commonly reported low oral bioavailability of natural products limits their effectiveness via systemic administration. To address these limitations, emerging delivery strategies, including nanoparticles, liposomes, hydrogels, and other ocular formulations, have shown considerable potential to enhance ocular bioavailability and retention, although their clinical translation remains under active investigation.

Second, the inherent limitations of natural product research should also be considered. Many studies rely on small sample sizes and lack independent validation, which may affect the reliability and generalizability of the findings. Furthermore, variations in extraction methods, formulation processes, and batch composition can lead to inconsistencies in active constituents and their biological activities, presenting challenges for standardization [104]. Such heterogeneity may contribute to discrepancies between preclinical and clinical outcomes and complicate direct comparisons across studies.

## 4. Conclusions

Current evidence suggests that several natural products have potential anti-inflammatory and immunomodulatory value. Among them, curcumin currently has relatively more clinical evidence, whereas RSV has attracted attention due to its broader pharmacological profile and emerging ocular formulations; however, their clinical utility in ocular diseases remains to be fully established and requires further validation in well-designed trials. Future studies should focus on further elucidating the underlying mechanisms, optimizing drug delivery strategies, and conducting more targeted basic and clinical research. Such efforts may contribute to the development of safer, more reliable, and cost-effective therapeutic approaches, while more accurately defining the therapeutic potential of these compounds and determining whether they may have a role as adjunctive or alternative treatment options in specific clinical contexts.

## Data Availability

No new data were created or analyzed in this study. Data sharing is not applicable to this article.
